# Shifting epidemiology of pancreatic cancer in Southeast Spain (1983-2018): emerging patterns in younger women and neuroendocrine neoplasms

**DOI:** 10.3389/fonc.2026.1717142

**Published:** 2026-02-17

**Authors:** Sandra M. Colorado-Yohar, Mónica Ballesta-Ruiz, Ricardo J. Vaamonde-Martín, Diego Salmerón, Javier Corral, Rafael Marcos-Gragera, Antonia Sánchez-Gil, María Dolores Chirlaque

**Affiliations:** 1 Department of Epidemiology, Murcia Regional Health Council, Murcia, Spain; 2 CIBER Epidemiología y Salud Pública (CIBERESP), Madrid, Spain; 3 Biomedical Research Institute of Murcia (IMIB), Murcia, Spain; 4 Research Group on Demography and Health, National Faculty of Public Health, University of Antioquia, Medellín, Colombia; 5 Health and Social Sciences Department, University of Murcia, Murcia, Spain; 6 Department of Medicine, Faculty of Medicine, University of Murcia, Murcia, Spain; 7 Servicio de Hematología y Oncología Médica, Centro Regional de Hemodonación, Hospital Universitario Morales Meseguer, Murcia, Spain; 8 CIBER Enfermedades Raras, Madrid, Spain; 9 Descriptive Epidemiology, Genetics and Cancer Prevention Research Group, Girona, Biomedical Research Institute (IdiBGi-CERCA), Girona, Spain; 10 Epidemiology Unit and Girona Cancer Registry, Oncology Coordination Plan, Department of Health Government of Catalonia, Catalan Institute of Oncology, Girona, Spain

**Keywords:** incidence, pancreatic cancer, pancreatic neuroendocrine neoplasm, Spain, survival population-based

## Abstract

**Background:**

Pancreatic cancer (PC) is among the most lethal cancers, with increasing incidence and poor survival worldwide. We examined population-based PC incidence and survival trends in the Spanish region of Murcia during 1983-2018.

**Methods:**

Population-based registry data were obtained. All primary invasive PC cases from 1983–2018 were included and classified as pancreatic neuroendocrine neoplasm (pNENs), non-pNENs and non-histologically-confirmed tumors. Age-specific and age-standardized incidence rates per 100,000 person-years (py) were calculated. Annual percentage changes (APCs) were estimated via joinpoint regression. Age-standardized net survival was calculated using the Pohar-Perme method. All results were stratified by sex, age, histological group and period.

**Results:**

In total, 3819 patients were diagnosed. The incidence rates in men increased from 11.2/100,000 py (1983–1988) to 21.8/100,000 py (2013–2018), and those in women increased from 7.2 to 15.2/100,000 py. The APC was more pronounced in women aged 15–44 years (APC =+ 5.2%, 95% CI = 1.5, 9.0) than in men (APC =+ 0.5, 95% CI=-1.5, 2.5). Among all confirmed cases, 93.3% were non-pNENs (APC = 5.4%, 95% CI = 4.9, 6.2), and 6.7% were pNENs (APC = 8.3%, 95% CI = 6.3, 13.6). Five-year survival rates were 5.1% (95% CI = 3.3, 7.0) in the 1990s and 11.4% (95% CI = 2.7, 13.1) in the 2010s (non-pNEN patients=7.4%, pNEN patients=57.5%).

**Conclusions:**

PC incidence increased in the Spanish region of Murcia, particularly among younger women and pNEN patients. Survival improved, doubling from the 1990s to the 2010s. These findings highlight the need to develop preventive strategies targeting high-risk populations, especially young women, while improving early PC diagnosis to continue increasing PC survival rates.

## Introduction

Pancreatic cancer (PC) is one of the most aggressive cancers worldwide and is the fourth leading cause of cancer-related mortality in Europe and the third leading cause in Spain. In 2022, there were more than half a million incident cases worldwide, with estimates that this number will double by 2040, causing more deaths than prostate, colon, and breast cancers combined (1). The incidence patterns of PC exhibit significant geographical variation, with socioeconomic factors playing a key role. Although several studies have reported sustained increases in PC across Western nations (2–4) important gaps persist particularly in long-term trend analyses (over three decades), demographic stratification by age and sex, and detailed data from Southern Europe. The prognosis of patients with PC is unfavorable, with five-year survival rates less than 6% (5, 6). Despite improvements in PC care, its prognosis remains unfavorable, underscoring the importance of prevention efforts.

Risk factors for PC include tobacco use, obesity, diabetes, chronic pancreatitis and heavy alcohol consumption and exposure to persistent organic pollutants (7–10). While this cancer is more common in men than in women (11), recent studies have shown an increase in incidence among women (particularly younger women) compared with men (4, 12).

Although several methods have been proposed for the early detection of PC (13), diagnosing it in the initial stages remains challenging, as it usually does not present with symptoms. Recent studies highlight the need to improve early detection through novel biomarkers and tailored therapeutic strategies (14). Furthermore, the US Preventive Services Task Force does not recommend routine screening for this cancer in asymptomatic adults (15).

PCs are broadly categorized into two main groups: pancreatic neuroendocrine neoplasm (pNENs) and non-pNENs. Non-pNENs, mainly adenocarcinomas are the most common and aggressive type, whereas pNENs are less common and have a better prognosis than non-pNENs (16).

Population-level monitoring of cancer incidence and survival provides essential indicators for cancer control (6, 17). Studies providing detailed and updated long-term trends in the incidence and survival of patients with PC are scarce (18). Therefore, the aim of the present study was to determine the population-based incidence and survival trends of PC over a long period in the Spanish Region of Murcia.

## Methods

### Study population

Data on newly diagnosed PC cases were obtained from the population-based Murcia Cancer Registry (RCM) located in southeastern Spain, which covers more than 1.5 million inhabitants in 2023 (19). The registry has been successfully operating since 1982 and is affiliated with the Murcia Regional Health Council Department of Epidemiology. The RCM is a member of the Spanish Network of Cancer Registries (Red Española de Registros de Cáncer—REDECAN; redecan.org/es/index.cfm) and the European Network of Cancer Registries (ENCR; www.encr.eu). The RCM participates in EUROCARE (https://www.iss.it/eurocare-il-progetto) and CONCORD (csg.lshtm.ac.uk), and its data are published in successive editions on ‘Cancer Incidence in Five Continents’, a reference publication edited by the International Agency for Research on Cancer (IARC, Lyon, France; ci5.iarc.fr) (20). Information on the sex and age of the cases were included as variable in the analysis and recorded by the Murcia Cancer Registry.

### Data collection

All registered patients with new invasive PC diagnosed within the 35-year period from January 1st, 1983, to December 31st, 2018, were included, except for those under 15 years of age, due to a lack of cases in the youngest age groups. All tumors, including PC, have topographic morphology and behavior codes according to the International Classification of Diseases for Oncology (ICD-O-3). Incident cases of primary invasive pancreatic neoplasms (C25.0 - C25.9), following the IARC-ENCR rules for the definition of primary cancers (21), were eligible for analysis. Tumors that were benign, *in situ*, or of uncertain nature were excluded. **Supplementary Figure 1** (Flowchart).

According to the Third Edition of the ICD-O, incident cases were classified into three histological groups: 1) histologically confirmed non-pNENs, with codes of 8010, 8012, 8020, 8022, 8031, 8050, 8070, 8074, 8140, 8144, 8211, 8230, 8260, 8310, 8440, 8441, 8450, 8452, 8453, 8470, 8480, 8481, 8490, 8500, 8523, 8550, 8560, and 8570; 2) histologically confirmed pNENs, with codes of 8013, 8042, 8150, 8151, 8152, 8153, 8240, 8246, and 8249; and 3) nonhistologically confirmed cases, with codes of 8000 and 8001. Sarcomas and lymphomas were not included in this analysis.

The survival data included complete follow-up data of the study participants from the date of diagnosis until December 31st, 2018. Vital status was ascertained on the basis of whether the participants were alive or dead at the end of follow-up (including the date of death) or were censored because of loss to follow-up or incomplete follow-up. Patients with complete follow-up were those with a known vital status (alive or dead) at one, three or five years after diagnosis. Multiple sources of information, including the National Death Index, the Social Security database, municipal censuses, medical records or hospital notes, primary care records, and pathology laboratories, were used to ascertain vital status. Data quality was verified using both the IARC check (22) and the EUROCARE rules (23). The variables used for this study were sex, age, date of birth, date of diagnosis, histology group, date of end of follow-up and vital status (alive, dead, censored, unknown).

The study was conducted according to the EU 2016/679 General Data Protection Regulation (GDPR) regarding the use of anonymized population data (24). All the data extracted and collected from the study database for incidence and survival analyses were anonymous; therefore, no ethical approval was required (25).

### Statistical methods

A descriptive analysis was conducted accounting for the total number of cases in the study period (1983 to 2018), both overall and stratified by sex and age groups (15-44, 45-54, 55-64, 65–74 and ≥75 years).

Age-adjusted standardized incidence rates (ASIRs) per 100,000 py and their 95% confidence intervals (95% CI) were also analyzed by sex, six-year period (1983–1988, 1989–1994, 1995–2000, 2001–2006, 2007–2012, 2013–2018), and histological group. ASIRs were calculated via the direct method (26) with the 2013 European Standard Population as the reference (27). To quantify changes in incidence trends for the entire study period (1983–2018) for the whole population and by age, sex, and histological group, we used joinpoint regression analysis of the ASIR data with their standard deviations. Annual percentage changes (APCs) and their 95% CIs were calculated, with each joinpoint marking a statistically significant change in the trend.

The number of joinpoints was set between 0 and 5, and the grid method and adjustment by permutation test (28) were selected, with an overall significance level of 0.05. The minimum number of data points from the beginning of the series was set at 4 years, and there were at least 5 years between two consecutive joinpoints.

Survival was calculated for patients diagnosed between 1990 and 2018. Age-standardized net survival (ASNS) analysis was performed for the study periods of 1990-1999, 2000-2009, and 2010-2018, which were stratified by sex, age, and histological group.

One (1y), three (3y), and five-year (5y) ASNS were calculated with the Pohar-Perme method (28), following the cohort approach and using International Cancer Survival Standards (ICSS) weights (29).

Analyses were performed using Stata/SE version 14, R packages (Epidemiology Tools 0.5-10.1), and the Joinpoint Regression Program, version 4.6 (30). P < 0.05 was considered to indicate statistical significance.

## Results

### Incidence trends

**Figure 1:**
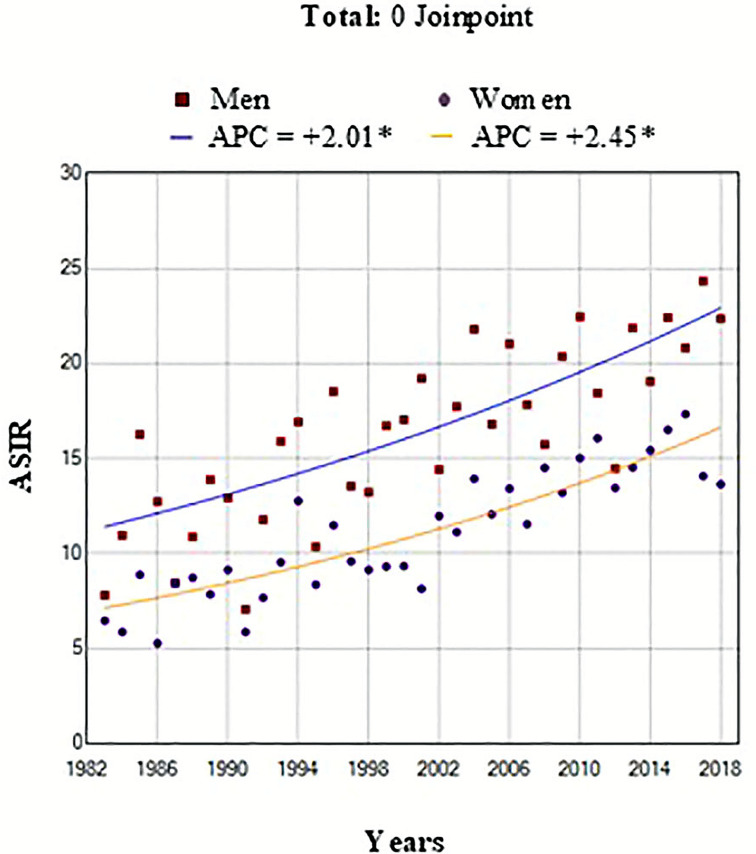
Trend change age standardized incidence rates (ASIR) of pancreatic cancer by both sexes and year of diagnosis in the Region of Murcia, Spain (1983-2018). ASIR: Age Standardized Incidence Rates per 100,000 person-years (2013 European Standard Population) for men (squared dots) and women (round dots). *Indicates that Annual Percent Change (APC) is significantly different from zero at the alpha = 0.05 level.

A total of 3819 patients (54% men) with PC were included in the analyses during the study period after excluding cases under 15 years of age, sarcomas, and lymphomas. Microscopic verification was confirmed in 61.4% of all PC patients. The mean age of the patients was 69.7 years (SD 12.4). Overall, the ASIR for both men and women doubled over the study period (1983–2018). In men, it increased from 11.2 to 21.8 per 100,000 py, with an APC of 2.0% (95% CI = 1.4, 2.6), whereas in women, it rose from 7.2 to 15.2 per 100,000 py, with an APC of 2.5% (95% CI = 2.0, 3.0), which was higher than that in men. These trends were statistically significant in both sexes. (**Figure 1**).

**Figure 2:**
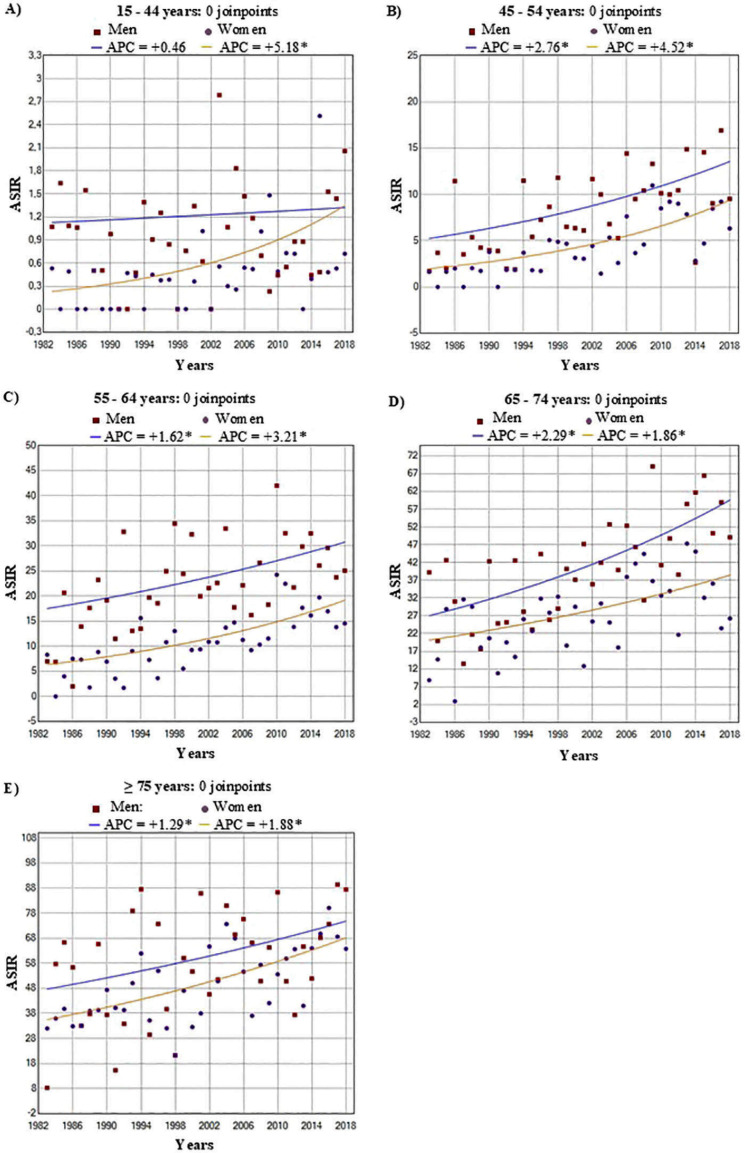
Trend change age standardized incidence rates (ASIR) of pancreatic cancer by sex age groups in the Region of Murcia, Spain (1983-2018). Age-specific trends in age-standardized incidence rates (ASIR) of pancreatic cancer by sex with joinpoint regression results. **(A)** 15–44 years, **(B)** 45–54 years, **(C)** 55–64 years, **(D)** 65–74 years, and **(E)** ≥75 years. Points represent observed ASIR values; lines represent fitted trends. Annual percent change (APC) is shown for men and women; * indicates statistically significant APC.

The incidence rates were higher in men than in women and increased with age, being lowest in the 15–44 years age group and highest in those aged ≥75 years. Young women presented a significant increase, with an APC of 5.2% (95% CI = 1.5, 9.0) in the 15-44-year age group and 4.5% (95% CI = 2.8, 6.3) in the 45-54-year age group, compared with lower APCs in men in the same age range, with 0.5% and 2.8%, respectively (**Table 1**, **Figure 2**).

**Table 1:** Age standardized incidence rates (ASIR) of pancreatic cancer by sex and period in the Region of Murcia, Spain. (1983-2018). (n= 3,819).

	1983 -1988				1989-1994				1995-2000				2001-2006				2007-2012					2013-2018					*1983-2018*		
	Cases	ASIR	95% CI		Cases	ASIR	95% CI		Cases	ASIR	95% CI		Cases	ASIR	95% CI		Cases		ASIR	95% CI		Cases		ASIR	95% CI		*APC (%)*	*95% CI*	
Men																													
Total	146	11.2	9.3	- 13.4	201	13.2	11.3	- 15.3	274	14.9	13.1	- 16.9	395	18.5	16.7	- 20.6	457		18.2	16.5	- 20.0	602		21.8	20.1	- 23.7	***2.0***	*1.4*	- 2.6
Age group																													
15-44	13	1.1	0.7	- 1.7	7	0.6	0.3	- 1.0	13	0.9	0.6	- 1.3	29	1.3	1.0	- 1.7	17		0.7	0.5	- 0.9	29		1.1	0.8	- 1.5	***0.5***	*-1.5*	- 2.5
45-54	15	4.6	3.7	- 5.6	14	4.5	3.7	- 5.6	27	7.7	6.7	- 8.9	40	9.1	8.1	- 10.2	60		10.6	9.6	- 11.7	75		11.3	10.3	- 12.3	***2.8***	*1.3*	- 4.2
55-64	33	11.5	10.0	- 13.1	59	18.8	17.1	- 20.8	76	25.6	23.5	- 27.9	74	22.8	20.9	- 24.9	104		26.4	24.5	- 28.4	123		27.7	25.9	- 29.7	***1.6***	*0.5*	- 2.7
65-74	44	27.8	24.7	- 31.1	65	30.4	27.6	- 33.4	87	33.4	30.8	- 36.2	128	45.0	42.1	- 48.1	132		45.7	42.8	- 48.8	177		57.4	54.2	- 60.8	***2.3***	*1.4*	- 3.1
≥75	41	43.3	36.7	- 51.0	56	53.7	47.0	- 61.4	71	46.5	41.3	- 52.3	124	68.6	62.5	- 75.2	144		59.3	54.6	- 64.4	198		72.8	68.1	- 77.8	***1.3***	*0.2*	- 2.4
Women																													
Total	121	7.3	6.0	- 8.8	168	8.8	7.5	- 10.3	214	9.5	8.3	- 10.9	308	11.8	10.5	- 13.2	425		14.0	12.7	- 15.4	508		15.2	13.9	- 16.6	***2.5***	*2.0*	- 3.0
Age group																													
15-44	2	0.3	0.1	- 0.6	2	0.2	0.0	- 0.4	4	0.3	0.1	- 0.5	9	0.4	0.3	- 0.7	20		0.8	0.6	- 1.1	17		0.8	0.5	- 1.1	***5.2***	*1.5*	- 9.0
45-54	3	1.2	0.8	- 1.8	7	2.1	1.6	- 2.8	12	3.6	2.9	- 4.4	18	4.2	3.5	- 5.0	42		7.9	6.9	- 8.7	42		6.6	5.8	- 7.4	***4.5***	*2.8*	- 6.3
55-64	15	4.8	3.9	- 5.8	26	7.6	6.5	- 8.8	27	8.2	7.1	- 9.5	41	11.8	10.5	- 13.2	64		15.4	14.0	- 16.9	76		16.4	15.1	- 18.0	***3.2***	*2.0*	- 4.4
65-74	41	19.6	17.5	- 22.0	47	18.6	16.6	- 20.7	84	27.1	25.0	- 29.5	83	25.0	23.0	- 27.2	116		35.0	32.6	- 37.5	123		34.9	32.6	- 37.3	***1.9***	*0.7*	- 3.0
≥75	60	35.6	31.4	- 40.4	86	46.4	41.7	- 51.4	87	36.9	33.3	- 40.8	157	58.8	54.4	- 63.4	183		52.7	49.2	- 56.4	250		64.8	61.1	- 68.6	***1.9***	*1.1*	- 3.0

ASIR, Age Standardized Incidence Rates per 100,000 person-years (2013 European Standard Population).

APC, Annual Percentage Change, calculated using joinpoint analysis by period and sex.

95% CI, 95% confidence interval.Bold values indicate APC (Annual Percentage Change), calculated using joinpoint analysis by period and sex.

The ASIRs of PC were also analyzed according to histological group. Non-pNEN cases constituted the majority (93.3%), with an ASIR of 7.9 (95% CI = 7.6, 8.2) per 100,000 py. Within this group, adenocarcinomas NOS remained the predominant subtype; however, its proportion declined over time, with a relative and absolute increase in infiltrating ductal carcinoma of pancreatic cancer IDC cases, particularly after 2000 (**Supplementary Figure 2**). Whereas pNEN cases were less common (6.7%), with an ASIR of 0.5 (95% CI = 0.4, 0.6) per 100,000 py. Non-histologically confirmed cases made up 38.6% of the total cases, with an ASIR of 5.8 (95% CI = 5.5, 6.1) per 100,000 py (**Table 2**).

**Table 2:** Age standardized incidence rates (ASIR) of pancreatic cancer by sex and age groups according to histological group in the Region of Murcia, Spain. (1983-2018).

	Overall PC					Non-pNEN					PNEN					Non-histologically-confirmed				
	Cases	%	ASIR	95% CI		Cases	%	ASIR	95% CI		Cases	%	ASIR	95% CI		Cases	%	ASIR	95% CI	
Total	3,819		14.2	13.8	- 14.7	2,188	93.3	7.9	7.6	- 8.2	157	6.7	0.5	0.4	- 0.6	1,474	38.6	5.8	5.5 - 6.1
Men	2,075	54.3	17.1	16.3	- 17.8	1,244	56.9	9.7	9.1	- 10.2	92	58.6	0.7	0.5	- 0.8	739	50.1	6.7	6.2 - 7.2
Women	1,744	45.7	11.7	11.2	- 12.3	944	43.1	6.3	5.9	- 6.7	65	41.4	0.4	0.3	- 0.5	735	49.9	5.0	4.6 - 5.3
Age group																				
15-44	162	4.2	0.9	0.8	- 1.0	109	5.0	0.6	0.5	- 0.7	33	21.0	0.2	0.1	- 0.2	20	1.4	0.1	0.1 - 0.2
45-54	355	9.3	6.8	6.1	- 7.5	280	12.8	5.3	4.7	- 6.0	29	18.5	0.5	0.3	- 0.7	46	3.1	0.9	0.6 - 1.1
55-64	718	18.8	16.8	15.6	- 18.1	514	23.5	12.0	11.0	- 13.1	37	23.6	0.9	0.6	- 1.1	167	11.3	3.9	3.3 - 4.5
65-74	1,127	29.5	34.1	32.1	- 36.1	738	33.7	22.3	20.7	- 23.9	30	19.1	0.9	0.6	- 1.2	359	24.4	10.9	9.8 - 12.0
≥75	1,457	38.2	55.1	52.1	- 57.8	547	25.0	20.0	18.3	- 21.7	28	17.8	1.0	0.6	- 1.4	882	59.8	33.9	31.7 - 36.2

ASIR: Age Standardized Incidence Rates per 100,000 person-years (2013 European Standard Population).

PNEN: Pancreatic neuroendocrine neoplasm.

95% CI: 95% confidence interval.

Sarcomas were excluded, n= 5.

**Figure 3:**
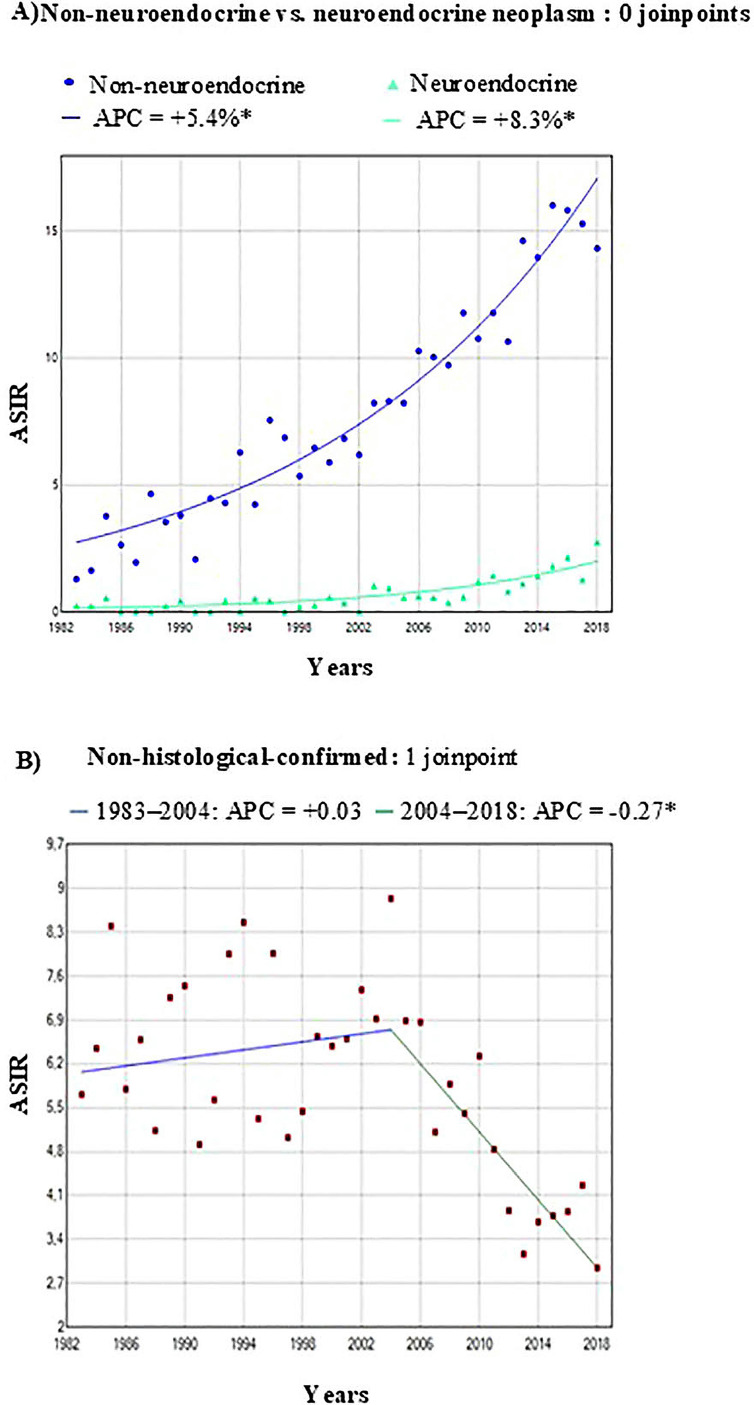
Trend and joinpoint analysis of age standardized incidence rates (ASIR) of pancreatic cancer by histological groups in the Region of Murcia, Spain (1983-2018). **(A)** Histology-specific trends. Blue dots: non-neuroendocrine pancreatic neoplasm; green triangles: neuroendocrine pancreatic neoplasm. **(B)** Non-histological-confirmed: Red squares = annual ASIR. The segmented regression depicts the trend change: blue line = pre-joinpoint trend. Green segment = post-joinpoint trend. ASIR per 100,000 person-years, standardized to the 2013 European Standard Population. Symbols show observed annual rates, solid lines show the best-fitting joinpoint model. *Indicates that Annual Percent Change (APC) is significantly different from zero at the alpha = 0.05 level.

An increasing trend in incidence was observed for both histological groups throughout the study period, with a significantly higher APC for pNENs (8.3%) than for non-pNENs (5.4%). With respect to tumors without histological confirmation, the joinpoint analysis revealed an APC of +0.03% per year from 1983 to 2004 no statistically significant. However, from 2004 to 2018, there was a statistically significant reduction in incidence rates, with an APC of -0.27% per year (**Figure 3**).

### Survival trends

The ASNS rates at one (1y), three (3y), and five (5) years after diagnosis decreased significantly over time, halving from 25.2% (95% CI = 23.7%, 26.7%) at 1y to 11.1%, (CI: 10%, 12.2%) at 3y, and further declining to 8.3% (95% CI = 7.3%, 9.4%) at 5y after diagnosis. Men had slightly higher survival rates than women did: 1 y (26.2% *vs.* 23.9%), 3 y (11.7% *vs.* 10.3%), and 5 y (8.7% *vs.* 7.9%). Survival rates decline with increasing age. Patients aged 15–44 years had the highest survival rates (54% at 1 y; 33.9% at 3 y; and 30% at 5 y). However, for those over 75 years of age, the rates were the lowest (13.9% at 1 y, 5.8% at 3 y, and 4.6% at 5 y). Survival rates improved over time. For patients diagnosed between 1990 and 2018,1y survival rates increased from 17.5% to 31.2%, 3 y survival rates rose from 6.9% to 14.6%, and 5 y survival rates improved from 5.1% to 11.4%. Survival rates also varied by histological type. Patients with non-pNENs had very low survival rates, with 28.1% at 1 y, 10.6% at 3 y, and 7.4% at 5 y. In contrast, those with pNENs had significantly higher survival rates: 79.0% at 1 y, 61.5% at 3 y, and 57.5% at 5 y (**Table 3**, **Figure 3**). Cases without microscopic verification had the poorest survival outcomes, with rates of 13.1% at 1 y, 5.4% at 3 y, and 3.8% at 5 y. Although the proportion of cases with microscopic verification increased steadily from the 1989–1994 period across all age groups and in both sexes reaching nearly 100% in most groups this improvement was not observed in the 75-year and older group. In this group, the percentage of cases with microscopic verification did not surpass 56% among women and 62% among men. Furthermore, among individuals aged 75 years and older, the proportion of cases identified solely through death certificates (DCOs) remained consistently higher than that of microscopically verified cases until the 2007–2012 period, when it began to decline and fall below the proportion of microscopically verified cases (**Supplementary Figure 3**).

**Table 3:** Age-standardized net survival at one, three and five years after diagnosis of pancreatic cancer by sex, age groups, period of diagnosis and histological groups in the Region of Murcia, Spain (1990-2018).

	Age-standardized Net Survival (95% CI)											
	1 Year				3 Years				5 Years			
	N	Rate (%)	95% CI		N	Rate (%)	95% CI		N	Rate (%)	95% CI	
Total	3,311	25.2	23.7	- 26.7	818	11.1	10.0	- 12.2	347	8.3	7.3	- 9.4
Sex												
Men	1,802	26.2	24.1	- 28.2	463	11.7	10.2	- 13.3	199	8.7	7.3	- 10.1
Women	1,509	23.9	21.8	- 26.1	355	10.3	8.7	- 11.9	148	7.9	6.3	- 9.5
Age group												
15-44	139	54.0	45.8	- 62.3	75	33.9	26.1	- 41.7	47	30.0	22.4	- 37.7
45-54	324	41.5	36.1	- 46.8	134	19.9	15.6	- 24.3	64	14.2	10.4	- 18.0
55-64	627	32.8	29.1	- 36.4	204	13.7	11.0	- 16.4	84	10.5	8.1	- 13.0
65-74	982	25.0	22.3	- 27.8	242	9.9	7.9	- 11.8	91	6.5	4.9	- 8.1
≥ 75	1,239	13.9	11.9	- 15.9	163	5.8	4.4	- 7.3	61	4.6	3.0	- 6.3
Period of diagnosis												
1990 -1999	617	17.5	14.5	- 20.6	106	6.9	4.8	- 8.9	40	5.1	3.3	- 7.0
2000 -2009	1,135	21.0	18.6	- 23.4	234	8.5	6.8	- 10.2	91	5.8	4.2	- 7.4
2010 -2018	1,559	31.2	28.9	- 33.6	478	14.6	12.8	- 16.4	216	11.4	9.7	- 13.1
Histological group												
Non-pNEN	2,025	28.1	26.1	- 30.1	561	10.6	9.3	- 12.0	206	7.4	6.2	- 8.6
PNEN	146	79.0	72.2	- 85.7	114	61.5	53.2	- 69.7	86	57.5	48.5	- 66.4
Non-histologically-confirmed	1,140	13.1	11.1	- 15.1	143	5.4	4.0	- 6.8	55	3.8	2.4	- 5.2

N: The number of pancreatic cancer patients included in the survival analysis.

PNEN: Pancreatic neuroendocrine neoplasm.

95% CI: 95% confidence interval.

Survival curves for the main subgroups are provided in the **Supplementary Material** (**Supplementary Figure 4**).

## Discussion

In the present study, we analyzed data on 3819 patients with PC who were diagnosed between 1983 and 2018. The incidence was notably higher in men than in women, especially among older individuals. Furthermore, the global literature consistently reports that PC is associated with increasing age and male sex (11). Overall, the incidence of PC has markedly increased over the past three decades in the Region of Murcia, rising more than double from 1983–1988 to 2013-2018. Recent studies evaluating long-term incidence (2000-2008) trends, particularly in developed countries, have shown significant increases in PC incidence, which aligns with our findings (31–33). Some of the increase could be due to improvements in cancer registration coding and clinical diagnostic accuracy over time. Although our analysis excluded sarcomas and lymphomas and this distinction was not specified in some of the comparative studies, the impact on the results must be minimal given the very low incidence of these cancers.

The incidence rates among women under 55 years increased at a higher rate than those among men of the same age group. This group experienced a significant increase in incidence, in contrast with the oldest age group (≥75 years), where the annual increase was far less pronounced, possibly reflecting the competing risk effects of premature death due to other causes. These findings align with several recent studies, including one study in which national population data from the United States revealed a greater increase in the incidence of PC in women aged 15 to 34 years (APC = 2.36%) than in men in the same age group (APC = 0.62%) between 2001 and 2018 (4). The reasons for the increase in the ASIR in younger women aged 15 to 54 years are unclear. Several aspects should be considered. The absolute number of cases was low, with ASIRs ranging from 0.3 to 7.9 in patients younger than 55 years, and small variations can lead to larger fluctuations in the APC. Compared with other population groups, young women may have different exposure patterns to modifiable risk factors. Moreover, changes over time toward less favorable lifestyle related risk profiles in young adult women are plausible (34). However, these explanations are proposed as possible hypotheses, not as findings from our data. In this context, recent evidence on early-onset pancreatic cancer highlights the importance of smoking as a major risk factor, with a stronger statistical association observed in early-onset cases compared to later-onset (35). This finding reinforces the role the modifiable lifestyle factors, particularly tobacco exposure, in the increasing incidence among younger women. However, it does not fully explain why the rise is more pronounced in women, as some studies have not identified a clear sex-related difference in risk. This suggests that, beyond smoking, other factors such as sex-specific differences in exposure patterns, hormonal influences, or underlying biological mechanisms may also contribute to the observed trends. The overall, increase in incidence observed in our study could be partially attributed to an aging population, improved diagnostic capabilities or changing patterns of risk factors.

Non-pNEN types predominated over pNEN types, with a greater proportion of cases in men (56.9%) than in women (43.1%). Another population-based study conducted in a region of northern Spain also highlighted a greater incidence of non-pNENs than pNENs (33). Although pNENs accounted for only 7% of the total confirmed cases, their incidence significantly increased over the analyzed period, which is consistent with findings of other studies (36).

In several studies, the number of confirmed PC cases has been reported to be relatively low compared with that of other types of cancer. In this study, the proportion of cases with microscopic confirmation was 61.4%, which is higher than that reported in a study in Spain (52% and 56.3%) (33, 37) and similar to figures reported in European Latin countries and United States (38). However, comparisons should be performed cautiously given that the proportion of microscopically confirmed cases has increased worldwide with increasing years since diagnosis. Notably, microscopic confirmation and more specific typing of the cancer are both mandatory for access to the best adjuvant treatment and whenever there is surgical cancer resection, which is the only hope for long-term healing. Therefore, it is not surprising that patients with PC without microscopic confirmation had the worst prognosis in our study and others (39).

The absence of histological verification may reflect a subgroup of patients diagnosed with more advanced disease and limited therapeutic intervention. These cases are often diagnosed clinically or radiologically or late presentation, when surgical resection is no longer feasible. Microscopic confirmation involves, at least, the acquisition of cancer cells through fine needle aspiration; even this (not to mention the retrieval of a biopsy sample) is an invasive procedure and may be associated with an important risk for serious complications, given the location of the pancreas. Nearly half of pancreatic cancer cases are diagnosed at advanced ages, in the 7^th^ and 8^th^ decades of life, when comorbidities are frequent and, in many instances, may contraindicate an aggressive approach disease management, limiting any possible benefit from microscopic assessment.

The fact that the proportion of cases without histological confirmation is age-dependent and that a similar age pattern has been reported for the pNEN versus non-pNEN ratio (33, 40) may affect the accuracy of our estimates regarding the relative proportion of both cancer types. Nonetheless, to our knowledge, no population-based cancer registry (PBCR)–based study exploring this issue is completely free from the substantial increase in the lack of microscopic verification with advancing age. Restricting the analysis only to microscopically confirmed patients would likely introduce additional selection bias and reduce the representativeness of the results for the real underlying population, particularly among the oldest age groups. Regarding potential misclassification of benign tumors as pancreatic cancers due to the lack of microscopic verification, this possibility cannot be completely excluded. However, diagnoses without pathology are usually supported by clinical and radiological information, and the specificity of CT and MRI for pancreatic cancer has been reported to be high in the literature (often >90% and >95%, respectively) (41, 42).

Since 2004, there has been a notable and statistically significant decrease in the number of non-histologically confirmed cases. This decline suggests improvements in diagnostic practices, with more cases being confirmed histologically, although further progress is still needed.

Although the prognosis remains poor, survival has doubled from 1990 to 2018, reaching 11.4% at five years compared with 5.1%, which was higher in men than in women and decreased with increasing age, in line with what has been reported in the literature. The SUDCAN study, whose data were extracted from the EUROCARE-5 study, reported an age-adjusted standardized net survival (5y-ASNS) of 6% for period 2000-2004, lower than that reported in our study (37). Similarly, a study analyzing the overall evolution of relative survival rates for pancreatic cancer also reports a slight increase in survival since 2000, although it concludes that no significant progress has been made in overall survival (43). This is consistent with recent evidence have reported that therapeutic advance have mainly benefited patients with favorable prognoses, while outcomes for the lowest prognosis groups, nearly 40% of de cases, have seen little to no improvement, highlighting the need for better patient stratification and integrated care strategies (44). It is possible that the slight survival improvement observed in our study reflects not only therapeutic advances but also earlier diagnosis; however, in the absence of information on disease stage at diagnosis, the relative contribution of these factors remains unclear. Although our data do not include individual treatment details, the observed improvements coincide with the introduction of more effective systemic therapies, such as mFOLFIRINOX suggesting that treatment innovation may have contributed to the survival gains in our population. In addition, contextual factors such as improved nursing care, greater patient awareness, and access to private care might have modestly contributed to slightly improved outcomes, possibly by facilitating at an earlier stage. What is well established, however, is that low survival rates are partly attributed to the advanced tumor stage at diagnosis in most cases; only approximately 10% to 15% of patients are diagnosed with early-stage (stage I) and surgically resectable disease (45).

In 2020, Siegel’s study highlighted an overall trend of improved cancer survival rates due to advancements in early detection and treatment techniques (46). However, for PC, no major improvements in prognosis have been achieved, especially for non-pNENs (47). Neither advances in therapeutic approaches with new selective molecules against specific cellular targets nor earlier detection using new high-resolution imaging techniques in recent years have significantly improved PC prognosis (48, 49). In this context, recent Mendelian randomization (MR) studies offer a promising direction by identifying potential biomarkers for predicting pancreatic cancer and informing treatment strategies (50, 51).

The survival of patients with pNENs was significantly higher than that of patients with non-pNENs. These findings are in line with better survival rates for pNENs reported globally (52), as well as in populations in Spain (33). The biological behavior of pNENs leads to earlier symptomatic presentation and thus earlier detection than non-pNENs. PNENs also usually grow more slowly than other types of PC. Because of these factors, pNENs are also more likely to be resectable (53). Additionally, pNENs tend to respond better to chemotherapy and radiation therapy than other types of PC do (54). The 5-year survival rate for pNEN patients is approximately 50% for all stages combined, whereas the 5-year survival rate for non-pNEN patients is less than 10% (33). However, importantly, individual patient factors, such as age, overall health, and the stage of cancer, can also impact survival outcomes.

The lowest survival rates were observed in the cases without microscopic verification, with additional disparities in diagnostic access observed according to age. The absence of microscopic verification particularly among those aged 75 years and older may be influenced by the challenges associated with advanced age. This contributes to lower rates of diagnostic confirmation and may partly explain the poorer survival outcomes observed in this group.

The major strength of this research is that the results are based on data from a population-based cancer registry that adheres to high-quality international standards. Additionally, the study period of more than three decades allows for an accurate assessment of population incidence trends and survival rates, providing a valid analysis of the epidemiological trend of this tumor. The results of the Region of Murcia could be similar to those of other regions in Spain because of the similarities in the health care system and sociodemographic and lifestyle backgrounds.

The percentage of cases without microscopic confirmation was 38.6%, which may affect the calculation of incidence and survival by introducing potential classification biases. Additionally, the small number of cases in certain subgroups could reduce the statistical precision of the indicator calculated. The lack of information on the tumor stage at diagnosis (and detailed treatment) is another limitation, precluding stage adjusted survival analyses.

## Conclusion

In the Spanish region of Murcia, the incidence of pancreatic cancer increased significantly over three decades, particularly among younger women and in for pNENs. One, three and five-year survival improved over the study period, doubling at each time point. Survival in the pNEN group was much higher than in non-pNENs and non-histologically confirmed cases, underscoring the prognostic relevance of histological classification, especially in older individuals. While the overall burden of pancreatic cancer remains highest among older adults, where its impact in terms of mortality is most significant, the rise in incidence among young women is especially concerning. Enhanced clinical vigilance in younger patients especially women with persistent, nonspecific gastrointestinal symptoms may help reduce diagnostic delays. Targeted prevention in high-risk populations and improved early diagnosis are essential to achieve the goal of increasing pancreatic cancer survival rates.

## Data Availability

The raw data supporting the conclusions of this article will be made available by the authors, without undue reservation.

## Glossary

APCs: Annual Percentage Changes
ASIRs: Age-Standardized Incidence Rates
ASNS: Age-Standardized Net Survival
CI: Confidence Interval
CONCORD: Global Surveillance of Cancer Survival Programme
DCOs: Death Certificate Only Cases
ENCR: European Network of Cancer Registries
EU: European Union
EUROCARE: European Cancer Registry Based Study on Survival and Care of Cancer Patients
GDPR: General Data Protection Regulation
IARC: International Agency for Research on Cancer
ICD: International Classification of Diseases
ICD-O-3: International Classification of Diseases for Oncology, 3rd Edition
ICSS: International Cancer Survival Standards
non-pNEN: Non-Pancreatic Neuroendocrine Neoplasm
PC: Pancreatic Cancer
pNENs: Pancreatic Neuroendocrine Neoplasm
py: Person-Years
RCM: Murcia Cancer Registry
REDECAN: Red Española de Registros de Cáncer
SD: Standard Deviation
SUDCAN: SUivi Des CANcers.

## References

[1] Bray F Laversanne M Sung H Ferlay J Siegel RL Soerjomataram I . Global cancer statistics 2022: GLOBOCAN estimates of incidence and mortality worldwide for 36 cancers in 185 countries. CA: Cancer J Clin. (2024) 74:229–63. doi: 10.3322/caac.21834, PMID: 38572751

[2] Hu J-X Zhao C-F Chen W-B Liu Q-C Li Q-W Lin Y-Y . Pancreatic cancer: A review of epidemiology, trend, and risk factors. World J Gastroenterol. (2021) 27:4298–321. doi: 10.3748/wjg.v27.i27.4298, PMID: 34366606 PMC8316912

[3] Huang J Lok V Ngai CH Zhang L Yuan J Lao XQ . Worldwide Burden of, Risk Factors for, and Trends in Pancreatic Cancer. Gastroenterology. (2021) 160:744–54. doi: 10.1053/j.gastro.2020.10.007, PMID: 33058868

[4] Abboud Y Samaan JS Oh J Jiang Y Randhawa N Lew D . Increasing pancreatic cancer incidence in young women in the United States: A population-based time-trend analysis, 2001-2018. Gastroenterology. (2023) 164:978–989.e6. doi: 10.1053/j.gastro.2023.01.022, PMID: 36775072 PMC11364483

[5] Red Española de Registros de Cáncer; REDECAN . Estimaciones de la Incidencia de Cáncer en España, 2024 (2024). Available online at: https://redecan.org/es/proyectos/15/estimaciones-de-la-incidencia-del-cancer-en-espana-2023 (Accessed February 2, 2026).

[6] Guevara M Molinuevo A Salmerón D Marcos-Gragera R Carulla M Chirlaque M-D . Cancer survival in adults in Spain: A population-based study of the spanish network of cancer registries (REDECAN). Cancers. (2022) 14:2441. doi: 10.3390/cancers14102441, PMID: 35626046 PMC9139549

[7] World Cancer Research Fund/American Institute for Cancer Research . Diet, nutrition, physical activity and pancreatic cancer (2018). Available online at: https://www.wcrf.org/sites/default/files/Pancreatic-cancer-report.pdfecommendations-2018.pdf (Accessed February 2, 2026).

[8] Naudin S Li K Jaouen T Assi N Kyro C Tjonneland A . Lifetime and baseline alcohol intakes and risk of pancreatic cancer in the European Prospective Investigation into Cancer and Nutrition study. Int J Cancer. (2018) 143:801–12. doi: 10.1002/ijc.31367, PMID: 29524225 PMC6481554

[9] Molina-Montes E Van Hoogstraten L Gomez-Rubio P Löhr M Sharp L Molero X . Pancreatic cancer risk in relation to lifetime smoking patterns, tobacco type, and dose-response relationships. Cancer epidemiology Biomarkers prevention: Publ Am Assoc Cancer Research cosponsored by Am Soc Prev Oncol. (2020) 29:1009–18. doi: 10.1158/1055-9965.EPI-19-1027, PMID: 32051190

[10] Porta M Gasull M Pumarega J Kiviranta H Rantakokko P Raaschou-Nielsen O . Plasma concentrations of persistent organic pollutants and pancreatic cancer risk. Int J Epidemiol. (2022) 51:479–90. doi: 10.1093/ije/dyab115, PMID: 34259837 PMC9082788

[11] Ferlay J Colombet M Soerjomataram I Dyba T Randi G Bettio M . Cancer incidence and mortality patterns in Europe: Estimates for 40 countries and 25 major cancers in 2018. Eur J Cancer (Oxford England: 1990). (2018) 103:356–87. doi: 10.1016/j.ejca.2018.07.005, PMID: 30100160

[12] Cavazzani A Angelini C Gregori D Cardone L . Cancer incidence (2000-2020) among individuals under 35: an emerging sex disparity in oncology. BMC Med. (2024) 22:363. doi: 10.1186/s12916-024-03574-x, PMID: 39232785 PMC11376010

[13] Sakai Y Honda M Matsui S Komori O Murayama T Fujiwara T . Development of novel diagnostic system for pancreatic cancer, including early stages, measuring mRNA of whole blood cells. Cancer Sci. (2019) 110:1364–88. doi: 10.1111/cas.13971, PMID: 30742728 PMC6447845

[14] Moretti M Farina A Angeloni A Anastasi E . Emerging horizons on molecular and circulating biomarkers in pancreatic adenocarcinoma. Front Oncol. (2024) 14:1483306. doi: 10.3389/fonc.2024.1483306, PMID: 39575418 PMC11578827

[15] Owens DK Davidson KW Krist AH Barry MJ Cabana M Caughey AB . Screening for pancreatic cancer: US preventive services task force reaffirmation recommendation statement. JAMA. (2019) 322:438–44. doi: 10.1001/jama.2019.10232, PMID: 31386141

[16] Stang A Wellmann I Holleczek B Fell B Terner S Lutz MP . Incidence and relative survival of pancreatic adenocarcinoma and pancreatic neuroendocrine neoplasms in Germany, 2009-2018. An in-depth analysis of two population-based cancer registries. Cancer Epidemiol. (2022) 79:102204. doi: 10.1016/j.canep.2022.102204, PMID: 35777306

[17] Galceran J Ameijide A Carulla M Mateos A Quiros JR Rojas D . Cancer incidence in Spain, 2015. Clin Trans oncology: Off Publ Fed Spanish Oncol Societies Natl Cancer Institute Mexico. (2017) 19:799–825. doi: 10.1007/s12094-016-1607-9, PMID: 28093701

[18] Kirkegård J Bojesen AB Nielsen MF Mortensen FV . Trends in pancreatic cancer incidence, characteristics, and outcomes in Denmark 1980-2019: A nationwide cohort study. Cancer Epidemiol. (2022) 80:102230. doi: 10.1016/j.canep.2022.102230, PMID: 35901622

[19] Centro Regional de Estadística de la Región de Murcia . Padrón Municipal de Habitantes. Available online at: https://econet.carm.es/web/crem/inicio/-/crem/sicrem/PU_padron/sec0.html (Accessed February 2, 2026).

[20] Bray F Colombet M Mery L Piñeros M Znaor A Zanetti R . Cancer Incidence in Five Continents, Vol. XI (electronic version). (IARC Scientific Publication No. 166). Lyon: International Agency for Research on Cancer (2019).

[21] IACR-ENCR . International rules for multiple primary cancers. 3rd ed. Lyon: IACR (2004). Available online at: http://www.iacr.com.fr/images/doc/MPrules_july2004.pdf (Accessed February 2, 2026).

[22] Ferlay J Burkhard C Whelan S PD . Check Conversion Programs for Cancer Registries. (IARC/IACR Tools for Cancer Registries). Lyon, France: IARC (2005).

[23] De Angelis R Francisci S Baili P Marchesi F Roazzi P Belot A . The EUROCARE-4 database on cancer survival in Europe: data standardisation, quality control and methods of statistical analysis. Eur J Cancer (Oxford England: 1990). (2009) 45:909–30. doi: 10.1016/j.ejca.2008.11.003, PMID: 19128955

[24] Regulation General Data Protection. Regulation (EU) 2016/679 of the European Parliament and of the Council of 27 April 2016. Available online at: https://eur-lex.europa.eu/eli/reg/2016/679/oj (Accessed January 15, 2022).

[25] European Network of Cancer Registries . Guidelines on confidentiality in population- based Cancer Registration in the European Union. Lyon: European Network of Cancer Registries (ENCR) (2002).

[26] Breslow NE Day NE . Statistical methods in cancer research. Volume II–The design and analysis of cohort studies. Lyon, France: IARC scientific publications (1987) p. 1–406. 3329634

[27] Commission. E . Revision of the European Standard Population—Report of Eurostat’s Task Force. Luxembourg: Publications Office of the European Union (2013).

[28] Perme MP Henderson R Stare J . An approach to estimation in relative survival regression. Biostatistics (Oxford England). (2009) 10:136–46. doi: 10.1093/biostatistics/kxn021, PMID: 18599516

[29] Corazziari I Quinn M Capocaccia R . Standard cancer patient population for age standardising survival ratios. Eur J Cancer (Oxford England: 1990). (2004) 40:2307–16. doi: 10.1016/j.ejca.2004.07.002, PMID: 15454257

[30] Joinpoint Regression Program, Version 4.6.0.0 - April 2018; Statistical Methodology and Applications Branch, Surveillance Research Program NCI. No Title (2018). Available online at: https://surveillance.cancer.gov/joinpoint/.

[31] Gaddam S Abboud Y Oh J Samaan JS Nissen NN Lu SC . Incidence of pancreatic cancer by age and sex in the US, 2000-2018. JAMA. (2021) 326:2075–7. doi: 10.1001/jama.2021.18859, PMID: 34689206 PMC8543346

[32] Samaan JS Abboud Y Oh J Jiang Y Watson R Park K . Pancreatic cancer incidence trends by race, ethnicity, age and sex in the United States: A population-based study, 2000-2018. Cancers. (2023) 15:870. doi: 10.3390/cancers15030870, PMID: 36765827 PMC9913805

[33] García-Velasco A Zacarías-Pons L Teixidor H Valeros M Liñan R Carmona-Garcia MC . Incidence and survival trends of pancreatic cancer in girona: impact of the change in patient care in the last 25 years. Int J Environ Res Public Health. (2020) 17:9538. doi: 10.3390/ijerph17249538, PMID: 33352812 PMC7766657

[34] Keyes KM Jager J Mal-Sarkar T Patrick ME Rutherford C Hasin D . Is there a recent epidemic of women’s drinking? A critical review of national studies. Alcoholism Clin Exp Res. (2019) 43:1344–59. doi: 10.1111/acer.14082, PMID: 31074877 PMC6602861

[35] Rémond M Smolenschi C Tarabay A Gelli M Fernandez-de-Sevilla E Mouawia A . Clinical and molecular features of early onset pancreatic adenocarcinoma. Int J Cancer. (2024) 155:1969–81. doi: 10.1002/ijc.35135, PMID: 39146492

[36] Jiang Y Abboud Y Liang J Larson B Osipov A Gong J . The disproportionate rise in pancreatic cancer in younger women is due to a rise in adenocarcinoma and not neuroendocrine tumors: A nationwide time-trend analysis using 2001–2018 United States cancer statistics databases. Cancers. (2024) 16:971. doi: 10.3390/cancers16050971, PMID: 38473332 PMC10931165

[37] Bouvier A-M Bossard N Colonna M Garcia-Velasco A Carulla M Manfredi S . Trends in net survival from pancreatic cancer in six European Latin countries: results from the SUDCAN population-based study. Eur J Cancer prevention: Off J Eur Cancer Prev Organisation (ECP). (2017) 26:S63–9. doi: 10.1097/CEJ.0000000000000303, PMID: 28005607

[38] Saad AM Turk T Al-Husseini MJ Abdel-Rahman O . Trends in pancreatic adenocarcinoma incidence and mortality in the United States in the last four decades; a SEER-based study. BMC Cancer. (2018) 18:688. doi: 10.1186/s12885-018-4610-4, PMID: 29940910 PMC6020186

[39] Huang L Jansen L Balavarca Y Babaei M van der Geest L Lemmens V . Stratified survival of resected and overall pancreatic cancer patients in Europe and the USA in the early twenty-first century: a large, international population-based study. BMC Med. (2018) 16:125. doi: 10.1186/s12916-018-1120-9, PMID: 30126408 PMC6102804

[40] Lei S Mao Y Yang Q Yan H Wang J . Trends in pancreatic cancer incidence, prevalence, and survival outcomes by histological subtypes: a retrospective cohort study. Gastroenterol Rep. (2025) 13:goaf030. doi: 10.1093/gastro/goaf030, PMID: 40207198 PMC11981714

[41] Toft J Hadden WJ Laurence JM Lam V Yuen L Janssen A . Imaging modalities in the diagnosis of pancreatic adenocarcinoma: A systematic review and meta-analysis of sensitivity, specificity and diagnostic accuracy. Eur J Radiol. (2017) 92:17–23. doi: 10.1016/j.ejrad.2017.04.009, PMID: 28624015

[42] Koelblinger C Ahmed B-S Goetzinger P Puchner S Weber M Sahora K . Radiology. Radiology (2011) 259:757–66. doi: 10.1148/radiol.11101189, PMID: 21436084

[43] Chen J Xiao Y-X Li Z-Y Zou Y-X Zhou X-H Zhang W . Global characteristics of pancreatic cancer survival: a comprehensive overview of survival analysis from cancer registration data. J Pancreatology. (2025) 8:307–17. doi: 10.1097/JP9.0000000000000216

[44] Kuijper SC Gehrels AM van der Geest LG Verhoeven RHA Koerkamp BG Molenaar IQ . Survival scenarios of patients with localized and metastatic pancreatic adenocarcinoma: A population-based study. Int J Cancer. (2025) 156:1726–35. doi: 10.1002/ijc.35267, PMID: 39614657 PMC11887001

[45] Blackford AL Canto MI Klein AP Hruban RH Goggins M . Recent trends in the incidence and survival of stage 1A pancreatic cancer: A surveillance, epidemiology, and end results analysis. J Natl Cancer Institute. (2020) 112:1162–9. doi: 10.1093/jnci/djaa004, PMID: 31958122 PMC7669234

[46] Siegel RL Miller KD Jemal A . Cancer statistics, 2020. CA: Cancer J Clin. (2020) 70:7–30. doi: 10.3322/caac.21590, PMID: 31912902

[47] McGuigan A Kelly P Turkington RC Jones C Coleman HG McCain RS . Pancreatic cancer: A review of clinical diagnosis, epidemiology, treatment and outcomes. World J Gastroenterol. (2018) 24:4846–61. doi: 10.3748/wjg.v24.i43.4846, PMID: 30487695 PMC6250924

[48] Halbrook CJ Lyssiotis CA Pasca di Magliano M Maitra A . Pancreatic cancer: Advances and challenges. Cell. (2023) 186:1729–54. doi: 10.1016/j.cell.2023.02.014, PMID: 37059070 PMC10182830

[49] Del Chiaro M Sugawara T Karam SD Messersmith WA . Advances in the management of pancreatic cancer. BMJ (Clinical Res ed). (2023) 383:e073995. doi: 10.1136/bmj-2022-073995, PMID: 38164628

[50] Zhong H Liu S Zhu J Xu TH Yu H Wu L . Elucidating the role of blood metabolites on pancreatic cancer risk using two-sample Mendelian randomization analysis. Int J Cancer. (2024) 154:852–62. doi: 10.1002/ijc.34771, PMID: 37860916 PMC10843029

[51] Zhong H Liu S Zhu J Wu L . Associations between genetically predicted levels of blood metabolites and pancreatic cancer risk. Int J Cancer. (2023) 153:103–10. doi: 10.1002/ijc.34466, PMID: 36757187

[52] Nikšić M Matz M Valkov M Marcos-Gragera R Stiller C Rosso S . World-wide trends in net survival from pancreatic cancer by morphological sub-type: An analysis of 1,258,329 adults diagnosed in 58 countries during 2000-2014 (CONCORD-3). Cancer Epidemiol. (2022) 80:102196. doi: 10.1016/j.canep.2022.102196, PMID: 35841761

[53] Kenney LM Hughes M . Surgical management of gastroenteropancreatic neuroendocrine tumors. Cancers. (2025) 17:377. doi: 10.3390/cancers17030377, PMID: 39941746 PMC11816225

[54] Benderli Cihan Y . Are PNETs radiotherapy resistant? Turkish J Surg. (2020) 36:238–9. doi: 10.5578/turkjsurg.4367, PMID: 33015571 PMC7515640

