# Supplementary material for: Shifting epidemiology of pancreatic cancer in Southeast Spain (1983-2018): emerging patterns in younger women and neuroendocrine neoplasms
Source: Front Oncol. 2026 Feb 17;16:1717142. doi: 10.3389/fonc.2026.1717142 (PMC12953114; doi:10.3389/fonc.2026.1717142)
Supplement: Supplementary file 1 [file Image1.pdf]

Supplementary Figure 1. Flowchart describing case identification and selection for incidence and survival analyses in the Murcia Cancer Registry.

Case identification:

Murcia Cancer Registry (population-based).

Inclusion criteria:

- Primary invasive pancreatic neoplasms.
- ICD-O-3 topography C25.0 – C25.9; primary cancer definition according to IARC-ENCR rules.

Exclusion criteria:

- Benign, in situ or uncertain behavior tumors.
- Lymphomas and sarcomas.
- No cases under 15 years were registred during the study period.

Incidence:

- Incidence trends (1983 – 2018).
- All eligible incident cases.

Survival:

- Diagnoses: 1990 – 2018; follow-up through 31 Dec 2018.
- Time from diagnosis to death or end of follow-up.
- Data sources: National Death Index, the Social Security database, municipal censuses, medical records or hospital notes, primary care records, and pathology laboratories.
- DCO cases were excluded from the main survival analyses.
- Age-standardized net survival (Pohar – Perme method).
