# Supplementary figures and images for: Shifting epidemiology of pancreatic cancer in Southeast Spain (1983-2018): emerging patterns in younger women and neuroendocrine neoplasms
Source: Front Oncol. 2026 Feb 17;16:1717142. doi: 10.3389/fonc.2026.1717142 (PMC12953114; doi:10.3389/fonc.2026.1717142)

Supplementary Figure 2

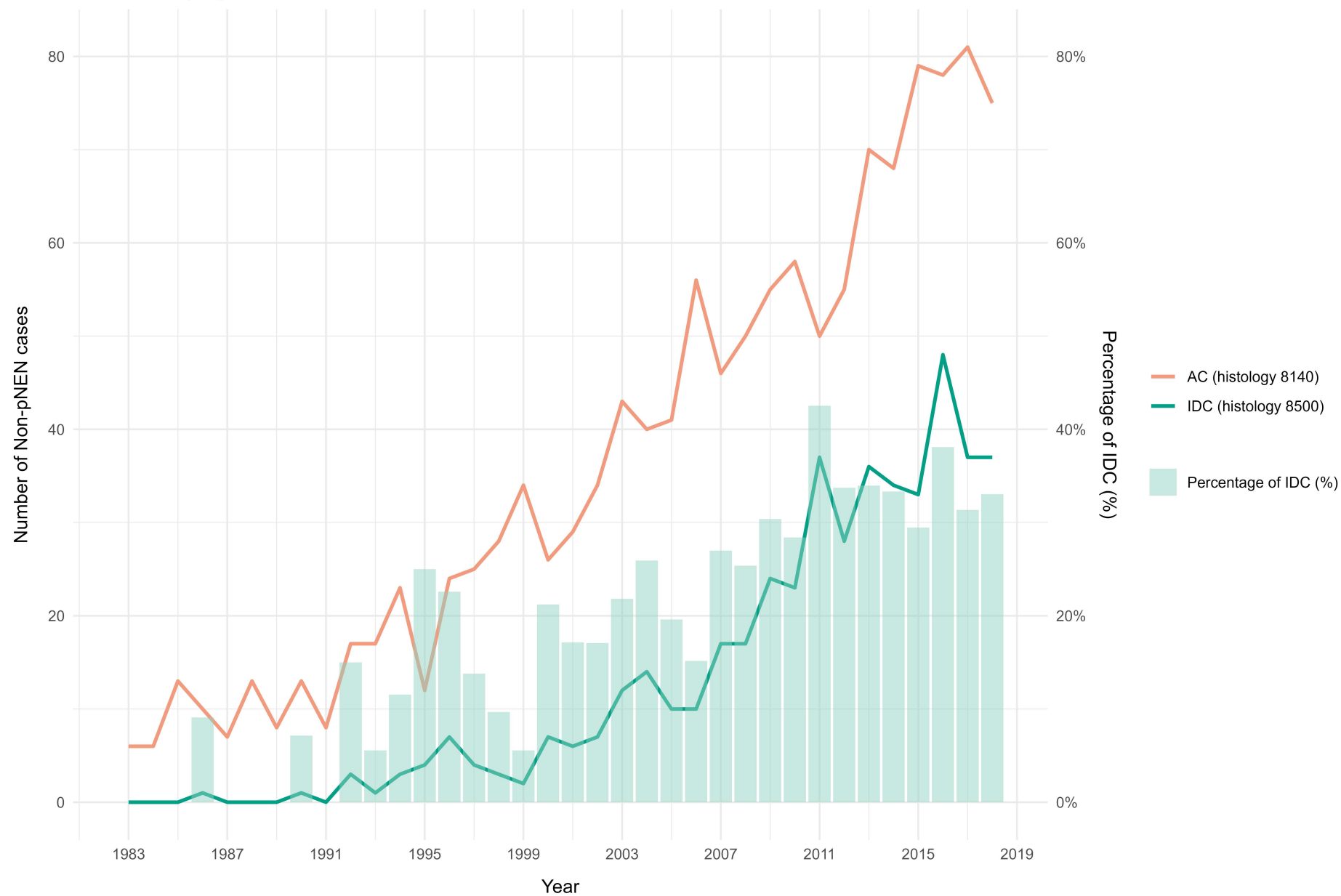

Supplement: Supplementary file 2 [file Image2.pdf]

Supplementary Figure 3

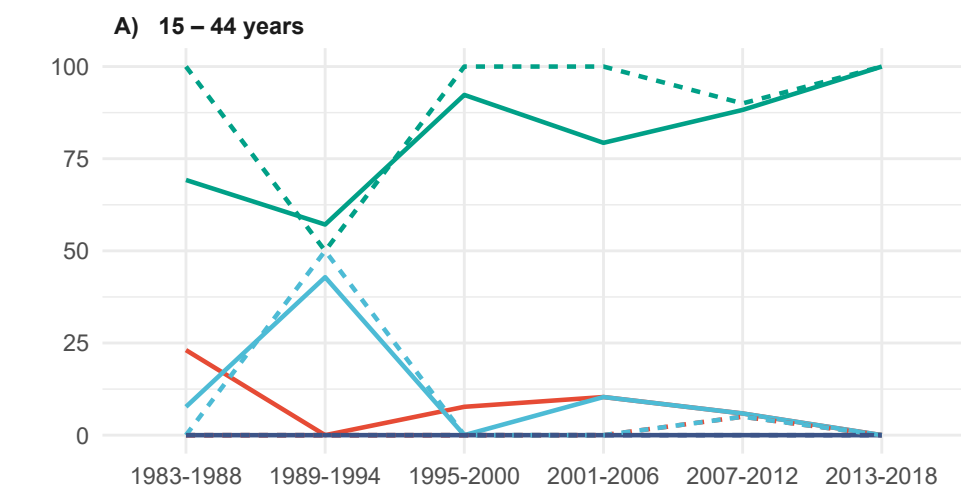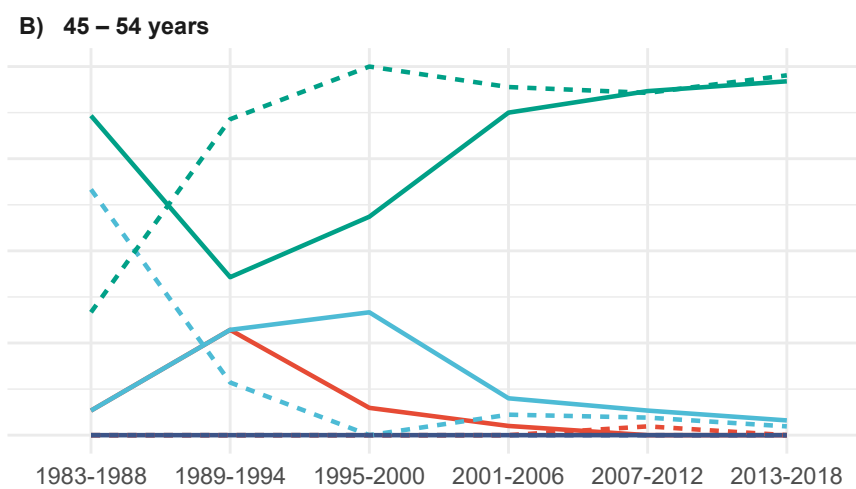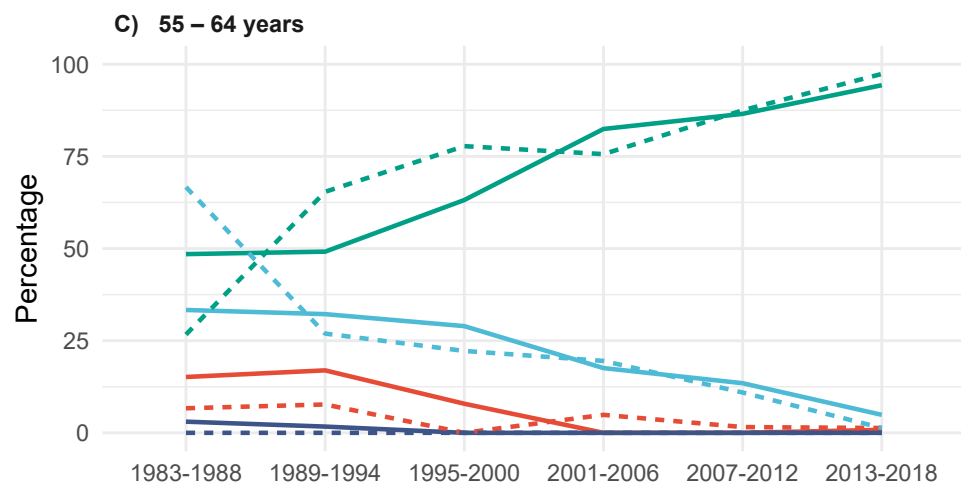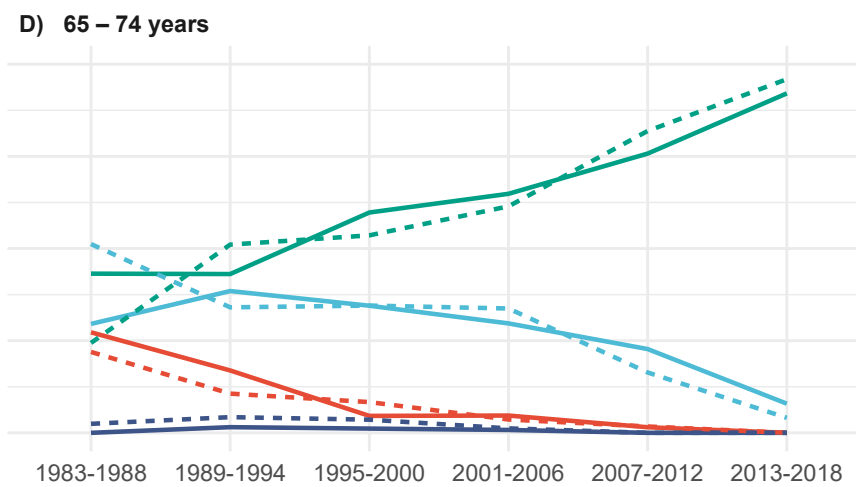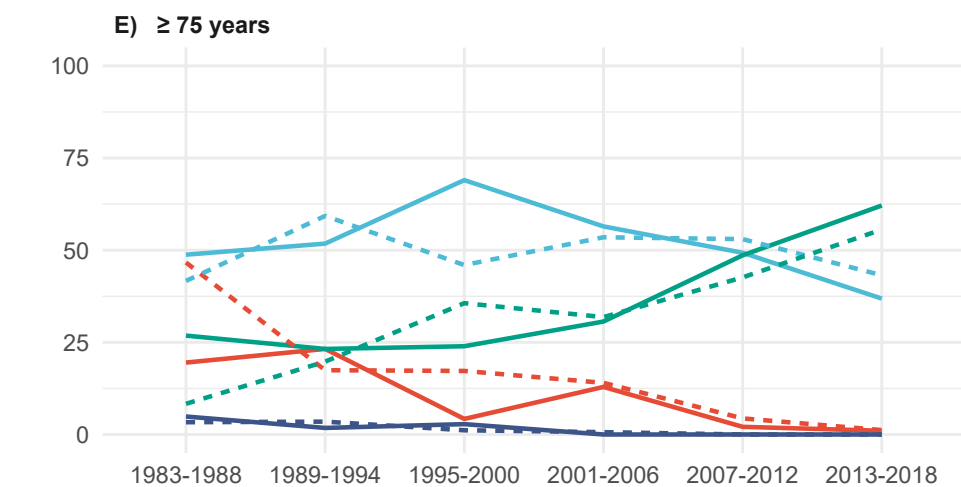

**Method**

- DCO
- No MV
- MV
- Unknown

**Sex**

- Men
- Women

Period

Supplement: Supplementary file 3 [file Image3.pdf]
