# Supplementary material for: Shifting epidemiology of pancreatic cancer in Southeast Spain (1983-2018): emerging patterns in younger women and neuroendocrine neoplasms
Source: Front Oncol. 2026 Feb 17;16:1717142. doi: 10.3389/fonc.2026.1717142 (PMC12953114; doi:10.3389/fonc.2026.1717142)

Supplementary Figure 4

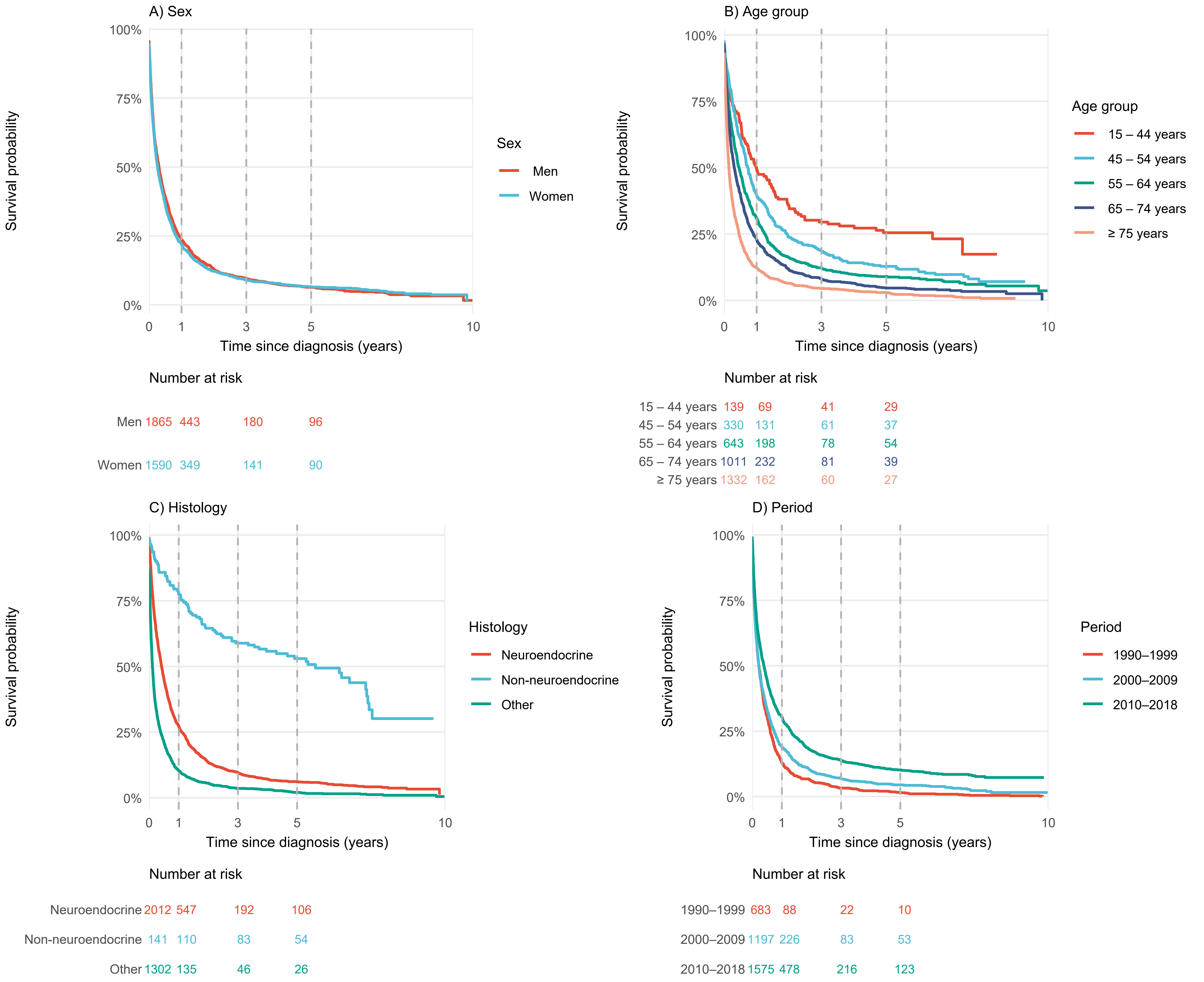

Supplement: Supplementary file 4 [file Image4.pdf]
